# Transcriptomic Analysis Revealed an Emerging Role of Alternative Splicing in Embryonal Tumor with Multilayered Rosettes

**DOI:** 10.3390/genes11091108

**Published:** 2020-09-22

**Authors:** Dina Hesham, Shahenda El-Naggar

**Affiliations:** Tumor Biology Research Program, Basic Research Unit, Research Department, Children’s Cancer Hospital Egypt-57357, Cairo 11441, Egypt; dina.hesham@57357.org

**Keywords:** ETMR, alternative splicing, LIN28A, ubiquitination, transcriptomics

## Abstract

Embryonal tumor with multilayered rosettes (ETMR) is an aggressive and rare pediatric embryonal brain tumor. Amplification of C19MC microRNA cluster and expression of *LIN28* are distinctive features of ETMR. Despite the increasing efforts to decipher ETMR, the biology remains poorly understood. To date, the role of aberrant alternative splicing in ETMR has not been thoroughly investigated. In the current study, a comprehensive analysis was performed on published unprocessed RNA-seq reads of tissue-matched ETMR and fetal controls datasets. Gene expression was quantified in samples using Kallisto/sleuth pipeline. For the alternative splicing analysis, STAR, SplAdder and rMATS were used. Functional enrichment analysis was subsequently performed using Metascape. The expression analysis identified a total of 3622 differentially expressed genes (DEGs) between ETMR and fetal controls while 1627 genes showed differential alternative splicing patterns. Interestingly, genes with significant alternative splicing events in ETMR were identified to be involved in signaling pathways such as ErbB, mTOR and MAPK pathways as well as ubiquitin-mediated proteolysis, cell cycle and autophagy. Moreover, up-regulated DEGs with alternative splicing events were involved in important biological processes including nuclear transport, regulation of cell cycle and regulation of Wnt signaling pathway. These findings highlight the role of aberrant alternative splicing in shaping the ETMR tumor landscape, and the identified pathways constitute potential therapeutic targets.

## 1. Introduction

Embryonal tumor with multilayered rosettes (ETMR) is a rare and aggressive pediatric embryonal brain tumor affecting infants and young children less than 4 years of age [1,2]. Despite intensive multimodal treatment, ETMR is a disease of poor prognosis with only 20% long-term survival [3,4,5,6,7,8,9]. ETMRs are mostly characterized by two distinctive molecular markers; amplification of Chr19q13.41 miRNA cluster (C19MC) [10] and high expression of *LIN28A*; a pluripotency factor and RNA-binding protein. Furthermore, the overexpression of a specific isoform of DNA methyltransferase 3 β (*DNMT3B*) was observed in ETMR [11]. High expression of *LIN28A* is implicated in neural development and pathogenesis of other advanced cancers [12]. Furthermore, the role of *LIN28A* in regulating splicing and gene expression programs has been investigated in cancer. For example, *LIN28A* was reported to cause significant isoform switches in genes involved in breast cancer biology [13]. In addition, LIN28A was demonstrated to bind to messenger RNAs (mRNAs) at specific motifs and regulates protein abundance of splicing regulators [14]. Despite the increasing efforts to decipher ETMR, an adequate understanding of the biology remains elusive. It is well established that alternative splicing (AS) is a key mechanism of post-transcriptional regulation of protein-coding genes that enables a single gene to produce multiple proteins [15].

Growing evidence has revealed that aberrant AS is one of the important hallmarks in cancer [16]. Through cellular plasticity that is offered by AS, cancer cells can produce certain protein isoforms favoring tumor growth and allow adaptation to their microenvironment [17,18]. Abnormal AS was also demonstrated to affect nearly all aspects of tumor biology, including cell cycle control, invasion, metastasis, angiogenesis, metabolism apoptosis, and drug resistance [19,20,21]. Many aberrant splicing events and variations in the abundance of alternatively spliced transcripts have been reported in different cancers including breast, pancreatic, liver, and multiple myeloma [22,23,24,25,26]. Thus, the potential role of AS in defining a new therapeutic target is being increasingly investigated. Nevertheless, the role of aberrant AS in ETMR remains largely unexplored.

In the current study, a comprehensive analysis was performed on published RNA-sequencing reads of ETMR and tissue-matched fetal controls from the Human Developmental Biology Resource (HDBR) datasets to identify and investigate the role of alternative splicing in ETMR. Our study showed that top up-regulated differentially expressed genes were involved in RNA splicing and processing, ubiquitination, and autophagy. Moreover, genes with AS events in ETMR were involved in developmental processes as well as cancer-related signaling pathways including ErbB, mTOR, and MAPK pathways. Finally, our work demonstrates the potential role of aberrant AS in the biology of ETMR.

## 2. Materials and Methods

### 2.1. Publicly Available RNA-Seq Data

Published unprocessed RNA-seq reads of nine ETMR samples were downloaded from NCBI’s Sequence Read Archive (SRA) (dataset accession number: SRP032476) [11], while the unprocessed RNA-seq reads of nine tissue-matched fetal controls from the Human Developmental Biology Resource (HDBR) were downloaded from ArrayExpress Archive (dataset accession number E-MTAB-4840) [27]. Both datasets were sequenced on an Illumina HiSeq 2000 (100-bp paired-end).

### 2.2. RNA-Seq Data Analysis

Quality control processes including adapter trimming, low-quality bases and short reads removal were performed on all the 18 fastq files using fastp software [28]. Links to QC reports are provided in Table S1. Gene expression was quantified using the Kallisto/sleuth pipeline. Read quantification was performed with Kallisto (v0.46.1), a pseudoalignment-based method to quantify RNA abundance at transcript-level in transcripts per million (TPM) counts [29]. Kallisto quant was utilized with the number of bootstraps set to 100 using ENSEMBL cDNA transcripts (Human assembly hg38 (GRCh38), release 94) for indexing. hierarchical clustering and principal component analysis (PCA) of the samples were performed and the plots are provided in Supplementary Materials (Figures S1 and S2). Downstream differential gene expression was performed using Sleuth R package (v0.30.0) [30] to leverage the bootstrap estimates of Kallisto and to output model-based, gene-level normalized TPM matrix. The normalized values were also corrected for potential batch effects due to RNA-seq data derived from two different sequencing core facilities. For each gene, both the likelihood ratio test (LRT) and Wald test (WT) were performed on the condition parameter to obtain their respective FDR-corrected *p*-values. Significant genes were those passing the two tests at a cutoff of false discovery rate (FDR) < 0.05. Plot_transcript_heatmap function in Sleuth package was utilized to visualize the cluster analysis. EnhancedVolcano R package was used to generate the volcano plot [31], which is a visual tool for displaying differentially expressed genes (DEGs) among overall gene expression levels. The Venn diagram was generated using InteractiVenn [32].

For the alternative splicing analysis, reads were mapped to the Human assembly hg38 using STAR. Two samples were excluded from the AS analysis (ETMR_9, as it was clustered with the controls and Control_3, as it had a low read count for the subsequent AS analysis). Identification and quantification of alternative splicing events (skipped exon, alternative 5′ splice site, and 3′ splice site, mutually exclusive exons, and retained intron) were carried out using both SplAdder [33] and rMATS (replicate multivariate analysis of transcript splicing) [34]. Only events that were detected by both AS tools in at least half of the ETMR samples compared to the controls at *p*-value < 0.05 were considered significant. IGV (Integrated Genomic Viewer) was utilized to visualize examples of genes with significant RI events in ETMR [35].

QC and alignment statistics are provided in Table S1.

### 2.3. Functional Enrichment and PPI Network Analysis

Functional enrichment analysis of differentially expressed and differentially spliced genes was subsequently performed using Metascape. All statistical values reported were corrected for multiple hypothesis testing by Benjamini–Hochberg FDR (*q*-values), and significant terms are chosen based on FDR < 0.05. STRING [36], and an online biological database and resource for known and predicted protein–protein interactions (PPIs) were used to construct a network of PPI of the DEG-encoded proteins with interaction score > 0.4 (medium confidence). Cytoscape software was then used for visualization of the PPIs network using “yFiles organic layout [37].

## 3. Results

### 3.1. Identification of Differentially Expressed Genes (DEGs) between ETMR and Fetal Normal Control

**Figure 1:**
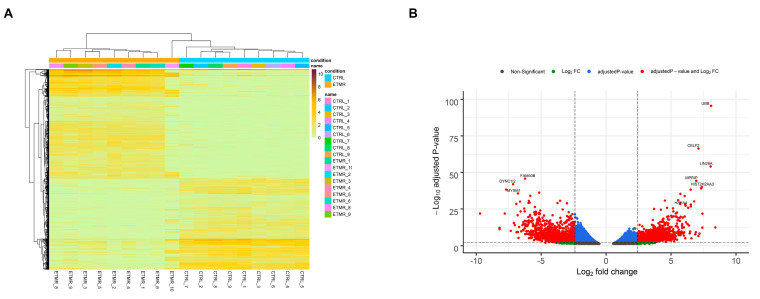
Overview of the differentially expressed genes (DEGs). (**A**) Heatmap of the differentially expressed transcripts. Each row read represents a single transcript, and each column represents a sample in each condition. (**B**) Volcano plot overviews the DEGs and highlighting the top up-regulated and down-regulated genes. The log2 (FC—fold change) is plotted on the *x*-axis, and the negative log10 adjusted *p*-value is plotted on the *y*-axis. The horizontal line represents the cutoff of the adjusted *p*-value (0.001) and the vertical lines represent the cutoff of the log2 FC (2.4 and −2.4).

After performing quality control and data normalization, a total of 5027 transcripts (3622 protein-coding genes) were identified by Sleuth. 2599 (2052 genes) were up-regulated and 2428 (1901 genes) were down-regulated, based on the cut-off criteria (adjusted *p*-value < 0.05 and log2 fold change (FC) between 2.4 and −2.4). Hierarchical cluster analysis demonstrated that the differentially expressed transcripts accurately distinguished ETMR samples from fetal control samples (Figure 1A). Volcano plot was generated to representDEGs with log2 FC scores and −log10 adjusted *p*-values (Figure 1B).

### 3.2. Enrichment Analysis of the DEGs

**Figure 2:**
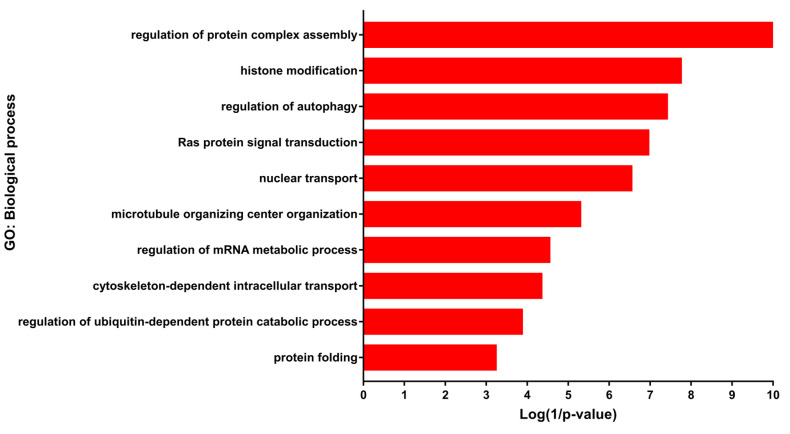
Gene ontology (GO) enrichment of differentially expressed genes (DEGs). Selected biological processes and their corresponding DEGs.

DEGs were mainly enriched in developmental processes including regulation of cell morphogenesis, regulation of neuron projection development, in addition to histone modification, regulation of autophagy, regulation of mRNA metabolic process, regulation of ubiquitin-dependent protein catabolic process and protein folding (Figure 2) (Table S1). According to the KEGG (Kyoto Encyclopedia of Genes and Genomes) pathway enrichment analysis, the DEGs were mainly enriched in Alcoholism, Axon guidance, Regulation of actin cytoskeleton, Cell cycle, Hedgehog signaling pathway, MAPK signaling pathway Proteoglycans in cancer and mTOR signaling pathway (Table S1). Most of the enriched biological processes and pathways were obtained from up-regulated genes, while the down-regulated genes made a lesser contribution.

### 3.3. PPI Network Analysis of Up-Regulated DEGs

**Figure 3:**
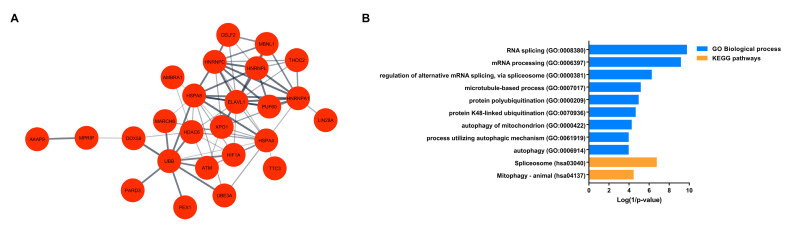
Enrichment and network analyses of up-regulated DEGs. (**A**) Protein–protein interaction (PPI) network of selected most up-regulated genes (nodes). The interactions (edges) line thickness indicates the strength of data support. (**B**) Enrichment analysis of the interacting up-regulated genes in the PPI network. Biological processes (blue) and KEGG pathways (orange) are plotted on the *y*-axis versus their Log (1/*p*-values) on the *x*-axis.

Network analysis of the up-regulated genes produced a PPI network –that is composed of 25 nodes (Table 1) and 68 edges (Figure 3A). PPI network was enriched in RNA splicing (FDR = 3.987 × 10^−06^), regulation of alternative mRNA splicing, via spliceosome (FDR = 0.00079), protein polyubiquitination (FDR = 0.0079), autophagy of mitochondrion (FDR = 0.025), autophagy (FDR = 0.039), and mitophagy (FDR = 0.015) (Figure 3B)(Table S2).

### 3.4. Alternative Splicing Events in ETMR

**Figure 4:**
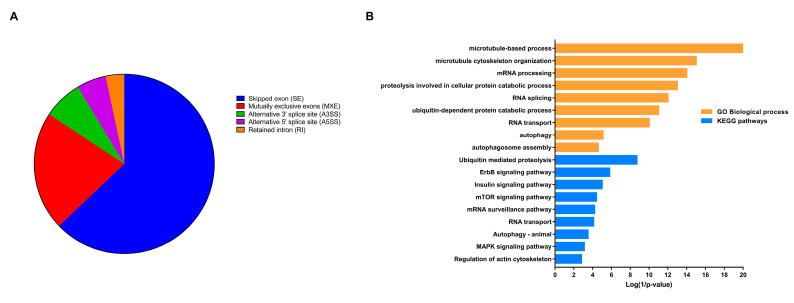
Overview of alternative splicing (AS) in embryonal tumor with multilayered rosettes (ETMR) and enrichment analysis of genes with AS events. (**A**). Pie chart showing genes’ count with different AS events. (**B**). Biological processes and KEGG pathways enriched by significant alternatively spliced genes.

A total of 1627 genes were detected to have significant AS events using both Spladder and rMATS tools in ETMR. The distribution of events was as follows: 1023 (63%) genes had SE, 349 (22%) genes had MXE, 115 (7%) genes had A3SS, 86 (5%) genes had A5SS, and 54 (3%) genes showed retained introns (Figure 4A). Interestingly, AS analysis also identified that one gene may undergo more than one type of AS event (Table S3). The significant alternatively spliced genes were enriched in microtubule-based process (FDR = 1 × 10^−16^), ubiquitin-mediated proteolysis (FDR = 6.3 × 10^−07^), RNA transport (FDR = 0.005), mRNA processing (FDR = 1 × 10^−11^), autophagy (FDR = 0.0007), as well as signaling pathways including ErbB signaling pathway (FDR = 0.00019), mTOR signaling pathway (FDR = 0.0025), MAPK signaling pathway (FDR = 0.031) (Figure 3B and Table S3).

### 3.5. Up-Regulated DEGs with AS Events

**Figure 5:**
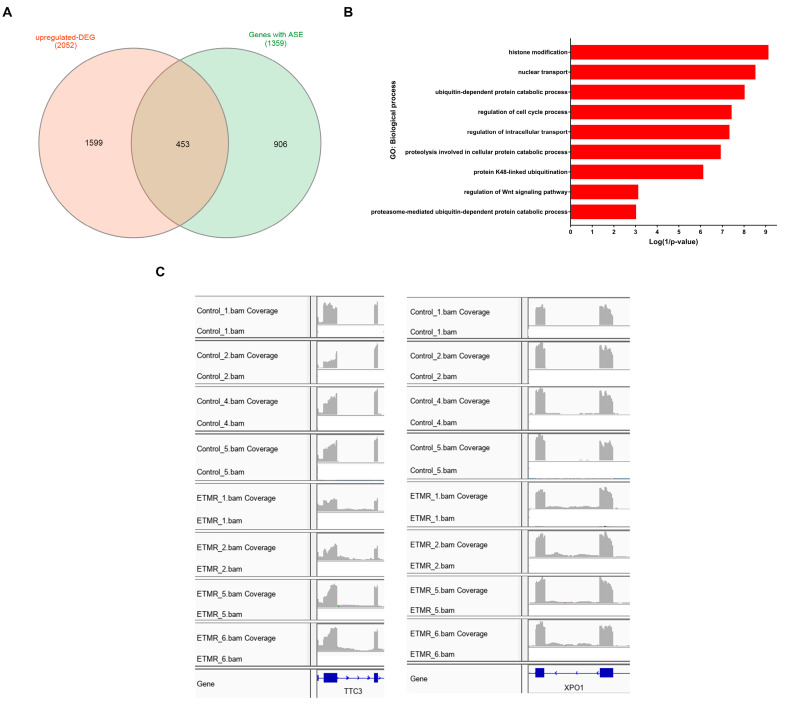
Enrichment analysis and examples of up-regulated differentially expressed genes (DEGs) with significant AS events. (**A**). Venn diagram showing common genes between DEGs and genes with significant AS events. (**B**). Biological processes are enriched by the up-regulated DEGs with significant AS events. (**C**). IGV(Integrated Genomic Viewer) plots demonstrate RIs in E3 ubiquitin-protein ligase (*TTC3*) and exportin-1 (*XPO1*) in ETMR compared to fetal control samples.

A total of 453 up-regulated DEGs were found to have significant AS events (Figure 5A) (Table S4). Enrichment analysis revealed that these genes were enriched in various biological processes and pathways like endocytosis (FDR = 5 × 10^−05^), histone modification (FDR = 1.2 × 10^−06^), nuclear transport (FDR = 3.1 × 10^−06^), ubiquitin-dependent protein catabolic process (FDR = 7.9 × 10^−06^), regulation of cell cycle process (FDR = 2.5 × 10^−05^), and regulation of Wnt signaling pathway (FDR = 0.03) (Figure 5B) (Table S4). Two examples of up-regulated genes that displayed intron retention in ETMR are Tetratricopeptide Repeat Domain 3 (*TTC3*) also known as E3 ubiquitin-protein ligase and exportin-1 (*XPO1*) (also referred to as CRM1) (Figure 5C). *TTC3* showed retention of intron 2, while *XPO1* showed retention of intron 12, in 8 out of the 9 ETMR cases.

## 4. Discussion

In the current study, we presented an overall transcriptomic analysis of ETMR to gain a better understanding of theirbiology and propose new therapeutic perspectives.Almost all human multi-exon genes are being regulated by AS. Cancer cells are capable of dynamically changing gene expression and favoring the expression of aberrant oncogenic splice variants to overcome stresses within the tumor microenvironment [38]. Abnormal AS is now regarded as a valuable indicator of carcinogenic processes and prognosis, as well as a potential target of treatment in several types of cancer [39,40,41,42,43,44].Not only is it a valuable diagnostic marker of ETMRs [45], *LIN28A* overexpression can be functionally significant as well. *LIN28A* was reported as a regulator of self-renewal capacity in cancer stem cells, cellular metabolism, and the cell cycle through binding and repression of let-7 microRNAs [12,46,47]. Our enrichment analysis of the PPI network of the top up-regulated protein-coding genes in ETMR identified their involvement in post-transcriptional regulatory pathways like mRNA processing and splicing, protein ubiquitination, and autophagy. Interestingly, an emerging role of *LIN28A* in carcinogenesis has been identified as being a modulator of alternative splicing and gene expression through regulation and interactions with splicing regulators like *HNRNPA1* [13,48]. Additionally, the exogenous *LIN28A* expression displayed widespread splicing changes utilizing splicing-sensitive microarrays [14]. In our study, *LIN28A* and splicing factors like *PUF60*, *HNRNPC*, *HNRNPA1*, and *HNRNPL* showed increased expression compared to fetal controls. Furthermore, *AMBRA1* (the activating molecule in BECN1-regulated autophagy protein 1) was found to be up-regulated in ETMR. Autophagy-related genes like *AMBRA1* were shown to play a role in cell survival and chemotherapy resistance [49]. Moreover, heat shock proteins (HSPs) like *HSPA4* and *HSPA8* were also among the most up-regulated genes in ETMR. HSPs were demonstrated to be involved in protein quality control, the ubiquitin–proteasome system (UPS), endoplasmic reticulum (ER) associated with degradation and autophagy [48]. In addition, it has been previously reported that HSPA4 and HSPA8 were associated with poor prognosis in cancer [50,51]. It was also found that UBB was the most up-regulated gene in ETMR compared to the controls. Tian and colleagues showed that increased expression of UBB was important for cancer initiation and maintenance of the cancer stem cell state [52]. Interestingly, the knockdown of UBB via small interfering RNA led to inhibition of survival and proliferation of tumor cells by suppressing ubiquitination at multiple sites associated with cancer pathways, and by impeding the ability of tumor cells to overcome increased stress [53].

The link between aberrant AS and signaling pathways in cancer has been discussed before [20,54,55]. In our study, DEGs with altered splicing events in ETMR were enriched in survival signaling pathways such as mTOR, MAPK pathways as well as ubiquitin-mediated proteolysis and cell cycle. A previous study also demonstrated the involvement of overexpression of LIN28A in the activation of the mTOR pathway indicating that inhibitors targeting the IGF/PI3K/mTOR pathway could be a promising novel treatment of ETMR [47].

Nuclear export was among the biological processes enriched by the up-regulated having significant AS events. Interestingly, the exportin-1 (*XPO1*) gene was among the most up-regulated genes and displayed retained intron events in ETMR. XPO1 is one of the key mediators of nuclear export, which is a crucial step in intracellular signaling, and it is utilized by cancer cells to stimulate cell proliferation and evade apoptosis [56]. XPO1 was found to be up-regulated in several cancer types and was demonstrated to dysregulate intracellular localization of tumor suppressors and oncogenes, modulate autophagy, and contribute to tumor growth and progression [57,58]. Therefore, nuclear export inhibition is considered a potential prognostic marker and therapeutic target for cancer [59,60,61]. Hence, further studies should be executed to detect the presence of the *XPO1* protein in ETMR and to investigate the effect of the retained intron on its functions.

Finally, our efforts in this in silico study identified genes with altered AS events and their functional significance in ETMR and provided targets for the critical experimental validation. Due to the scarcity of ETMR cases, the sample size in our study was limited, and therefore, we recommend the establishment of multicenter-based studies for the experimental validation of this findings.

## 5. Conclusions

In conclusion, our study describes a potential underlying mechanism in the carcinogenesis of ETMR, which is altered AS. Further investigations are needed to validate the ETMR-specific splicing events, which can be a potential therapeutic target for this fatal embryonic tumor.

## Figures and Tables

**Table 1:** List of interacting up-regulated protein-coding genes.

Gene	log2FC	Adjusted *p*-Value	Description
*UBB*	8.085482	2.67 × 10^−96^	Polyubiquitin-B
*LIN28A*	8.048782	8.71 × 10^−55^	Protein lin-28 homolog A
*XPO1*	7.413878	1.61 × 10^−22^	Exportin-1
*CELF2*	7.108907	3.64 × 10^−67^	CUGBP Elav-like family member 2
*MPRIP*	6.943921	5.21 × 10^−45^	Myosin phosphatase Rho-interacting protein
*TTC3*	6.149392	1.34 × 10^−16^	E3 ubiquitin-protein ligase TTC3
*MARCH6*	6.124846	5.56 × 10^−28^	E3 ubiquitin-protein ligase MARCH6
*HSPA4*	6.113184	5.34 × 10^−28^	Heat shock protein family A member 4
*PEX1*	6.093165	1.01 × 10^−28^	Peroxisome biogenesis factor 1
*PARD3*	6.049788	1.30 × 10^−20^	Partitioning defective 3 homolog
*AKAP9*	6.028525	2.06 × 10^−07^	A-kinase anchor protein 9
*HIF1A*	6.020426	6.87 × 10^−10^	Hypoxia-inducible factor 1-α
*THOC2*	6.000342	1.32 × 10^−07^	THO complex subunit 2
*PUF60*	5.996787	3.37 × 10^−13^	Poly(U)-binding-splicing factor PUF60
*ATM*	5.95952	2.46 × 10^−09^	Serine-protein kinase ATM
*HSPA8*	5.240461	0.000871	Heat shock cognate 71 kDa protein
*HNRNPC*	5.153226	3.94 × 10^−26^	Heterogeneous nuclear ribonucleoproteins C1/C2
*AMBRA1*	3.968858	0.001036	Activating molecule in BECN1-regulated autophagy protein 1
*HDAC6*	3.413792	2.43 × 10^−06^	Histone deacetylase 6
*UBE3A*	3.258943	0.000783	Ubiquitin-protein ligase E3A
*HNRNPA1*	3.215216	8.56 × 10^−07^	Heterogeneous nuclear ribonucleoprotein A1
*HNRNPL*	3.004006	0.015223	Heterogeneous nuclear ribonucleoprotein L
*MBNL1*	2.853727	0.000229	Muscleblind-like protein 1
*DDX58*	2.621277	0.011365	Probable ATP-dependent RNA helicase DDX58
*ELAVL1*	2.438159	3.69 × 10^−05^	ELAV-like protein 1

(FC = fold-change).

## Supplementary Materials

The following are available online at https://www.mdpi.com/2073-4425/11/9/1108/s1, Table S1: DEGs and enrichment analysis. Table S2: Enrichment analysis of the genes in the PPI. Table S3: Genes with AS events and enrichment analysis of DEGs with AS events. Table S4: Up-regulated genes with RI events and their enrichment analysis. Figure S1: Principal component analysis (PCA) of the ETMR and fetal control samples. Figure S2: Heatmap-based hierarchical clustering of the samples.
